# Effect of Topological Defects on Buckling Behavior of Single-walled Carbon Nanotube

**DOI:** 10.1007/s11671-010-9776-x

**Published:** 2010-09-05

**Authors:** Ali Reza Ranjbartoreh, Guoxiu Wang

**Affiliations:** 1 School of Mechanical Engineering, Faculty of Engineering, University of Technology Sydney, Office 3. 29, Building 4, Harris St, Broadway, Sydney, NSW 2007, Australia; 2 Department of Chemistry and Forensic Science, University of Technology, Sydney, NSW 2007, Australia

**Keywords:** Molecular dynamic simulation, Buckling, Single-walled carbon nanotube (SWCNT), Stone–Wales (SW) defect, Single vacancy (SV) defect

## Abstract

Molecular dynamic simulation method has been employed to consider the critical buckling force, pressure, and strain of pristine and defected single-walled carbon nanotube (SWCNT) under axial compression. Effects of length, radius, chirality, Stone–Wales (SW) defect, and single vacancy (SV) defect on buckling behavior of SWCNTs have been studied. Obtained results indicate that axial stability of SWCNT reduces significantly due to topological defects. Critical buckling strain is more susceptible to defects than critical buckling force. Both SW and SV defects decrease the buckling mode of SWCNT. Comparative approach of this study leads to more reliable design of nanostructures.

## Introduction

Carbon nanotube (CNT) shows outstanding properties in almost all fields of technology. Strong covalent bonds between carbon atoms and unique honeycomb cells in cylindrical structure lead to its superior mechanical properties. CNTs have been widely used in composites [1], hydrogen storages [2], and other advanced technical applications [3,4]. Theoretical and experimental methods have been employed to study the mechanical properties of CNTs.

Theoretical approaches found much higher amounts of strength and stability as they assumed a perfect structure for nanotubes; while, in practice, it is almost impossible to find a CNT without structural defects and imperfections. Some experimental methods like Raman spectroscopy, electron spin resonance, and optical absorption spectroscopy have been employed to estimate defect's type and concentration on the surface of nanotubes [5].

Purification, acid treatment [6-8], and oxidation at high temperature [9,10] could cause defects on the structure of CNT; in addition, ball milling [11] and irradiation [12] have been intentionally utilized to make defects.

Defects have positive effects on the strength of nanocomposites as interfacial bonding sites, on storage of hydrogen as penetration and atomic storage sites [13-15], on transition of nanotubes from one diameter to another, and on Y junction in molecular electronic [16]. Achieving defects' advantages in such applications, it is necessary to consider their effects on mechanical behaviors of CNTs.

Effects of defects and imperfections on bending buckling behavior of nanotubes were considered by finite element and molecular dynamic simulation (MDS) methods [17]. Buckling behaviors of CNTs under axial compression have been studied by MDS but reported results are in contradiction with each other [18-20]. Continuum mechanics method has been successfully employed to predict the mechanical behavior of pristine nanotube [21-24] but influences of defect, chirality, and wall thickness on mechanical behavior of CNT cannot be investigated precisely by continuum mechanics.

Differences between local and average properties in macrostructures are serious issues, these differences are even more critical in nanostructures. There are few reports about the impact of defects on mechanical properties of nanotubes and most of them studied the tensile properties of defected CNTs; whereas, nanotubes could be subjected to compressive loads in many applications such as composites, hydrogen storages, etc. In addition, CNTs are very susceptible to compression [25].

In practice, topological defects exist on the structure of nanotubes. Obtaining more reliable results, their reducing effects on mechanical properties of CNTs should be considered accurately. Therefore, study of critical buckling forces and strains of single-walled carbon nanotube (SWCNTs) with structural defects like Stone–Wales (SW) and single vacancy (SV) defects can shed the light on mechanical properties of nanotubes under compression.

Comparative investigation into buckling behaviors of pristine and defected armchair and zigzag SWCNTs with various lengths and radiuses under axial compression by MDS method is the subject of this research, which has not been discussed previously.

## Modeling

SWCNTs with different lengths and radiuses (Table 1) and wall thickness of 0.66 Å have been generated [26]. Large-scale atomic/molecular massively parallel simulator (LAMMPS) code, which is an open source code, was employed to simulate the responses of nanotubes to external loads. This code uses LJ unitless quantities [27,28]. Bond energy of 4.89866 eV [29] and bond length of 1.42 Å [30] were utilized to convert the units from LJ to SI. Axial compressive load has been applied on atoms at one end when atoms at another end have been clamped to make cantilever boundary condition.

**Table 1:** Dimensions of armchair and zigzag SWCNTs

	Radius(Å)	Length(Å)
Armchair (3,3)	2.064	13.554
Armchair (4,4)	2.735	13.546
Armchair (5,5)	3.409	13.543
Armchair (6,6)	4.085	13.541
Zigzag (3,0)	1.216	15.121
Zigzag (4,0)	1.597	15.017
Zigzag (5,0)	1.982	14.982
Zigzag (6,0)	2.370	14.963

Buckling behaviors of SWCNTs have been displayed by visual molecular dynamics (VMD) program [31]. VMD have been employed for the visualization of deformations and post-processing of results such as condensation of SWCNTs under axial compression.

Pressure and strain of SWCNT obtained as below

(1) $$\varepsilon =\frac{\delta l}{l_{0}}$$

(2) $$P=\frac{F}{A}$$

A=πdt

where *P* is the pressure, *F* is the force, *d* is the diameter, *t* is the thickness (0.66 Å), *A* is the cross sectional area, *ε* is the strain, *δl* is the variation of length, and *l*_0_ is the initial length.

## Results and Discussion

**Figure 1:**
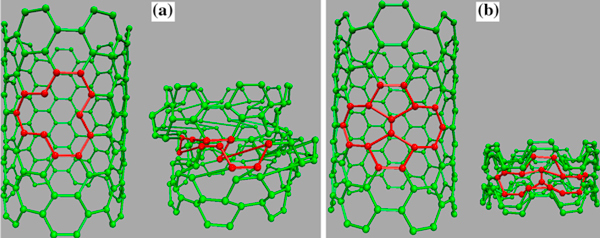
**Buckling behavior of armchair SWCNT with single vacancy (a) and Stone–Wales (b) defects**.

**Figure 2:**
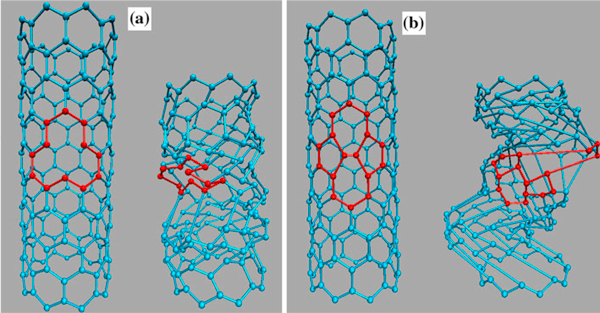
**Buckling behavior of zigzag SWCNT with single vacancy (a) and Stone–Wales (b) defects**.

SW defect is a topological defect due to 90° rotation of C–C bond and formation of two pentagonal and two heptagonal cells. Another topological defect is SV defect, which is made by omission of one atom from honeycomb structure of CNT (Figures 1, 2).

The fraction of defected area to surface of tube in small nanotubes is relatively high; as a result, defect has high damaging effect on structural stability of CNT [32,33]. Presented results in Table 2 indicate that negative influence of defects weakens gradually by growth of the radius.

**Table 2:** Critical buckling force (F), pressure (P), and strain (ε) of pristine (P) SWCNTs and SWCNTs with Stone–Wales (SW) and single vacancy (SV) defects

	F_P_ (nN)	P_P_ (GPa)	ε_p_	F_SW_(nN)	P_SW_(GPa)	ε_SW_	F_SV_(nN)	P_SV_(GPa)	ε_SV_
(3,3)	0.740	8.644	0.477	0.376	4.392	0.102	0.309	3.614	0.083
(4,4)	1.048	9.239	0.718	0.452	3.981	0.129	0.491	4.325	0.157
(5,5)	0.864	6.112	0.488	0.786	5.560	0.453	0.535	3.785	0.187
(6,6)	1.003	5.924	0.658	0.942	5.562	0.625	0.552	3.258	0.199
(3,0)	0.329	6.522	0.135	0.256	5.086	0.105	0.226	4.478	0.063
(4,0)	0.365	5.513	0.167	0.162	2.439	0.042	0.273	4.124	0.093
(5,0)	0.329	3.999	0.136	0.248	3.016	0.099	0.279	3.389	0.097
(6,0)	0.365	3.715	0.167	0.176	1.786	0.049	0.289	2.949	0.106

Hexagonal cells in the structure of CNTs reveal a great condensation under compression, such structure empower CNTs against yielding under compression; nevertheless, they lose their stability due to buckling when axial pressure exceeds the critical buckling pressure. Critical buckling pressure of CNT is much lower than its yielding stress, particularly when defects exist on its structure.

Defects change the stress distribution of nanotube under axial compression and due to stress concentration at defects' locations, nanotube shows deflection in lateral direction and buckles. Tubes tend to undergo buckling in the lowest mode to reduce the overall system energy [34]. Location of defects affects the buckling mode, whereas defects' type and size govern the critical buckling force and strain of SWCNT. In general, defects have negative influence on the stability of CNT; they reduce the critical bucking force and strain of nanotube under compression [35-37].

**Figure 3:**
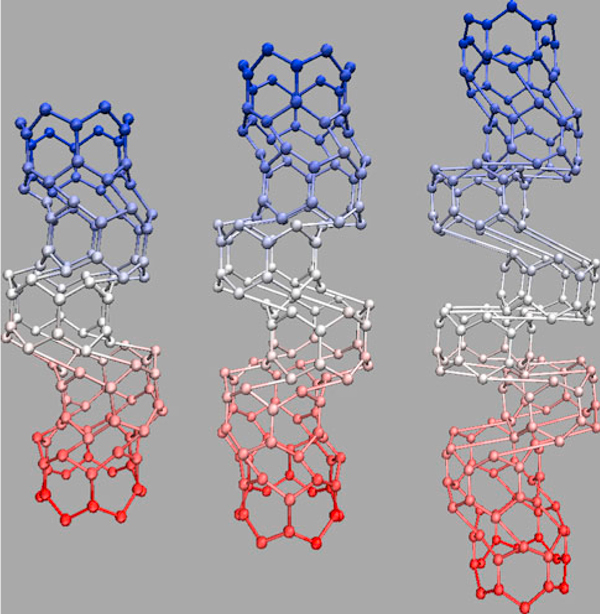
**Increase in buckling mode with rising of the length**.

Second mode of buckling in axial direction of zigzag SWCNTs does not change with growth of radius but it increases gradually by rising of the length (Figure 3).

Buckling mode of armchair SWCNTs decreases as the radius increases, while it increases as the length increases (i.e. armchair nanotubes with higher aspect ratios (length/radius) show higher buckling modes).

Both SV and SW defects caused buckling under lower loads and in lower modes. Location of defects has an essential effect on buckling mode; moreover, failure of the structure starts from defects' locations; these are consistent with predicted results of Cao and Chen [35] about bifurcation modes of SWCNTs.

Mielke et al. [38] reported higher strain limits for shorter nanotubes. They noted more destructive effect of vacancy defects than SW defects on the stability of CNTs. They found 26% reduction in failure stress and up to 100% reduction in failure strain caused by vacancy defects on the structure of CNTs.

Sammalkorpi et al. [39] confirmed that mechanical properties of defected nanotubes depend on the chirality; they found 50% lower critical strain for defected SWCNT. Liew et al. [40] declared that CNT bundles show higher critical buckling force and lower critical buckling strain by growth of the radius. Cao and Chen [34] found critical buckling strain of 0.11–0.13 for zigzag SWCNTs, which is consistent with presented results in Table 2 and Figure 5.

Obtained results in Table 2 indicate that omission of one atom has generally more severe effect than SW defect on axial stability of armchair SWCNTs but zigzag SWCNTs show opposite trend; SW defects have mostly higher influence on their critical buckling force, pressure, and strain.

As shown in Figure 1b, SW defect in armchair SWCNT occurs by replacement of a horizontal bond with an axial bond. On the other hand, rotation of an axial bond to horizontal mode causes SW defect in zigzag SWCNT (Figure 2b), while resistance of nanotube against buckling depends on its axial stability. That is why SW-defected zigzag SWCNT is such susceptible to axial compression.

Critical buckling forces of pristine SWCNTs with presented dimensions in Table 1 show fluctuations with variation of radius. Shell-like behavior of these nanotubes can be ascribed to their low aspect ratios, which was declared by Ranjbartoreh et al. [22]. However, rising the radius, critical buckling pressure of SWCNT decreases gradually due to increase in the cross-section area.

Critical buckling forces and strains of armchair and zigzag SWCNTs with SV defects increase as the radius grows. Armchair SWCNTs with SW defects show the same trend, while zigzag nanotubes with SW defects and pristine SWCNTs reveal harmonic fluctuations in their trends. Such behavior can be attributed to relatively local effect of SW defect and condense atom arrangement of zigzag nanotube, which does not let it to be compressed as much as armchair type.

**Figure 4:**
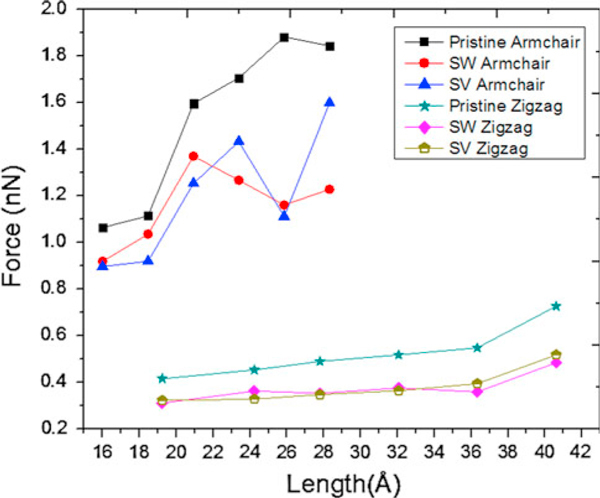
**Critical buckling forces versus length of pristine, Stone–Wales (SW)-defected, and single vacancy (SV)-defected armchair (6,6) and zigzag (6,0) SWCNTs**.

**Figure 5:**
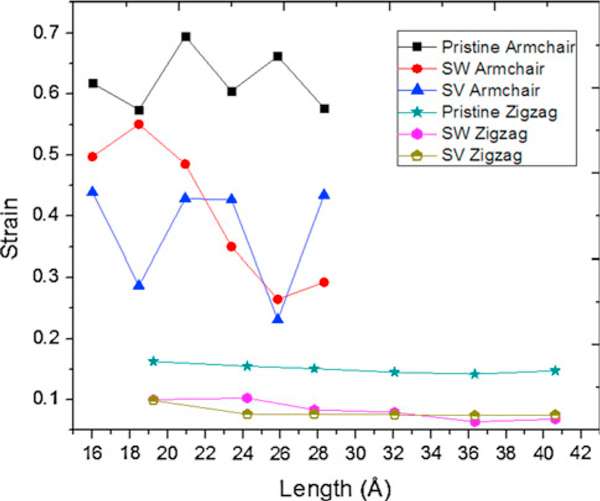
**Critical buckling strains versus length of pristine, Stone–Wales (SW)-defected, and single vacancy (SV)-defected armchair (6,6) and zigzag (6,0) SWCNTs**.

Increasing the length of zigzag SWCNTs from 19.23 to 40.61 nm, critical buckling forces of pristine, SW-defected, and SV-defected zigzag SWCNTs rise from 0.41 to 0.72 nN, 0.31 to 0.48 nN, and 0.32 to 0.51 nN, respectively, while critical buckling strains of them decrease from 0.16 to 0.14, 0.1 to 0.06, and 0.1 to 0.07 (Figures 4, 5). Beam-like behaviors of zigzag SWCNTs are ascribed to their relatively high aspect ratios [17].

Increasing the length of armchair SWCNTs from 16 to 28.3 nm, pristine and defected armchair SWCNTs show fluctuations in their critical buckling forces and strains; similar trend was reported by Cao and Chen [35]. These fluctuations (i.e., shell-like behaviors) are due to their low aspect ratios [18]. It can be depicted from Figures 4 and 5 that critical buckling force and strain of SWCNTs decrease by 7.5–62% and 3.6–250% due to SW defects also those of SWCNTs decline by 13–69% and 25–286% owing to SV defects.

Reducing effect of defects on critical buckling force and strain of both armchair and zigzag nanotubes can be clearly realized from Table 2 and Figures 4 and 5. In general, armchair SWCNT can carry higher compression before its axial failure. Critical axial force of defected zigzag SWCNT is about 44–78% of pristine one and that of armchair type is 43–94% of pristine armchair SWCNT (Table 2).

Defects have more severe effects on critical strain than critical force of nanotubes. Critical buckling strains of SWCNTs with short radiuses drop dramatically due to SW and SV defects (Table 2).

Comparative style of this study describes the destructive effects of topological defects on the stability of SWCNT and leads to more reliable design calculation for nanostructures. Comparing critical properties of pristine and defected SWCNTs in this article provides correction factors to temperate obtained results of previous theoretical studies, which ignored the effects of topological defects on mechanical properties of CNTs.

## Conclusions

Buckling behaviors and critical axial forces, pressures, and strains of pristine and defected SWCNTs with different lengths, chiralities, and radiuses have been considered with MDS method.

Armchair nanotube shows higher strength under compression. Increasing the length, critical buckling force of pristine SWCNT increases, while this trend is inversed for critical buckling strain of pristine SWCNT.

Topological defects reduce the axial stability of SWCNTs, especially nanotubes with small radiuses. SV and SW defects significantly decrease the critical buckling forces and strains of SWCNTs.

Either SW or SV defects reduce the buckling mode of SWCNT. Buckling mode of zigzag SWCNT remains constant with variation of radius but it increases by rising of the length; whereas, armchair SWCNTs with higher aspect ratios show higher buckling modes.

Critical buckling strain is more vulnerable to defects than critical buckling force i.e., critical strain of defected SWCNT is about one-third of critical strain of pristine SWCNT.

Predicted results of theoretical articles that assumed a perfect structure for CNT can be moderated by presented results of this study.
